# Measurement of three‐dimensional changes in lip vermilion in adult female patients after orthodontic extraction: a retrospective longitudinal study

**DOI:** 10.1186/s13005-021-00263-5

**Published:** 2021-03-19

**Authors:** Lin-hui Shen, Tian-yi Xie, Ruo-ping Jiang, Yi-ran Jiang, Gui Chen, Tian-min Xu, Bing Han

**Affiliations:** 1 Third Clinical Division, Clinical Research Center for Oral Diseases & National Engineering Laboratory for Digital and Material Technology of Stomatology & Beijing Key Laboratory of Digital Stomatology, Peking University School and Hospital of Stomatology & National, Beijing, China; 2 Second Clinical Division, Research Center for Oral Diseases & National Engineering Laboratory for Digital and Material Technology of Stomatology & Beijing Key Laboratory of Digital Stomatology, Peking University School and Hospital of Stomatology & National Clinical, Beijing, China; 3 Department of Orthodontics, Peking University School and Hospital of Stomatology & National Clinical Research Center for Oral Diseases & National Engineering Laboratory for Digital and Material Technology of Stomatology & Beijing Key Laboratory of Digital Stomatology, 22 Zhongguancun South Avenue, Haidian District Beijing, 100081 PR China

**Keywords:** Lip vermilion, Three‐dimensional scanner, Soft tissue, Extraction treatment

## Abstract

**Background:**

3D facial scanning has changed the way facial aesthetic is evaluated and has numerous advantages for facial analysis. The specific relationship between lip vermilion morphological changes after orthodontic extraction treatment has not been fully explained. The objective of this study was to evaluate 3D morphological changes after orthodontic extraction treatment in lip vermilion of adult females with dentoalveolar protrusion using a structured light-based scanner.

**Methods:**

Forty-two female subjects (25.2 ± 1.9 years) were recruited as the treatment group; these patients had undergone extraction treatment and achieved better sagittal profiles. Twenty female subjects (25.5 ± 2.1 years) were enrolled in the non-treatment group; these patients did not require any orthodontic treatment. The follow up time for the treatment group was more than 24 months and for the non-treatment group was more than 12 months. 3D facial scans were captured using 3D CaMega. Six landmarks (Ls, Li, R.Chp, L.Chp, R.Ch, and L.Ch), three linear measurements (mouth height, philtrum width, and mouth width), and three area measurements (upper, lower, and total vermilion area) were measured. The spatial deviations of three volumetric measurements (upper, lower, and total vermilion) were constructed for quantitative analysis. Color-coded displacement map were constructed for visualization of the soft-tissue displacement as qualitative evaluation.

**Results:**

Mouth height and philtrum width decreased (-0.93 mm and − 1.08 mm, respectively) significantly (*p* = 0.008 and *p* = 0.027, respectively), and no significant (*p* = 0.488) change in mouth width was observed in the treatment group. The lower and total vermilion surface areas decreased (-51.00mm^2^ and − 69.82mm^2^, respectively) significantly (*p* = 0.003 and *p* = 0.031, respectively) in the treatment group, but no statistically significant (*p* = 0.752) change was detected in the upper vermilion. In the treatment group, significant retractions were observed in the color-coded displacement map, and three volumetric measurements of vermilion changed significantly (*p* = 0.012, *p* = 0.001 and *p* = 0.004, respectively). Significant differences were found between the treatment group and the non-treatment group in the linear, area and volumetric measurements.

**Conclusions:**

This study established a method for qualitative and quantitative evaluation of the lip vermilion. Significant 3D retraction of the lip vermilion after the extraction treatment was found, with morphological variation between upper and lower vermilion.

## Background

Facial aesthetics are important in social environments; thus, orthodontic treatment now places more emphasis on facial soft tissues, particularly the lips [1]. Malocclusion and procumbency of the lips are common chief complaints in the Asian population. Orthodontic extraction treatment is an effective way to relieve lip protrusion [2–5] because it can achieve better facial aesthetics as a straight profile with a mesocephalic face type and balanced facial appearance.

In the past, a reduction in lip protrusion following extraction of the premolars in patients with dentoalveolar protrusion was evaluated using two-dimensional (2D) imaging. Iared et al. reported that maxillary and mandibular lip distances were retracted 1.3 ± 1.8 mm and 1.2 ± 1.4 mm, respectively, after extraction [5]. The information provided by 2D imaging was limited in describing the border of the lip vermilion, and lip vermilion changes are three dimensional (3D). Therefore, morphological changes in the lip vermilion should be studied using 3D techniques. 3D facial imaging devices, such as stereophotogrammetry, laser scanning, and structured light scanning, have facilitated faster, non-invasive, and accurate measurements [6]. 3D measurements of the lips, including linear, area, and volumetric measurements, can be easily obtained and assessed using 3D analysis software with rapid development [6].

In facial soft tissues, the lip vermilion is a prominent feature due to its obvious soft tissue contours compared with facial skin [7]. Several studies have explored the general 3D facial structures of Asian adult female patients and highlighted significant morphological influences on the lip vermilion in patients who were considered more attractive than those who were considered average [7, 8]. Several studies have demonstrated significant differences in lip vermilion curvature and shape during orthodontic treatment using 3D facial scanning [9, 10]. However, the specific relationship between lip vermilion morphological changes after orthodontic extraction has not been fully explained, which makes the prediction of post-treatment vermilion morphology difficult. Thus, it is important to gain a better understanding of morphological changes in the lip vermilion when assessing the need for orthodontic extraction. The objective of the present study was to evaluate 3D morphological changes in the lip vermilion in adult females undergoing four premolar extractions using a structured light-based scanner.

## Methods

### Subjects

This was a retrospective longitudinal study. The study was approved by the Ethics Committee of Peking University School and Hospital of Stomatology (PKUSSIRB-201,948,110). The sample size of this preliminary study was determined according to historical studies (Solem RC et al. [11]: 24 subjects; Jung J et al. [12]: 32 subjects; Li H et al. [13]: 50 subjects).

The forty-two subjects (25.2 ± 1.9 years) in the treatment group were recruited from consecutive patients during their initial visit to the Department of Orthodontics, Peking University, School and Hospital of Stomatology, Beijing, China. In the treatment group, all the patients were treated using the same fixed appliances (0.022 × 0.028-inch bracket slot, Roth prescription; Xinya, Hangzhou, China) for at least 24 months.

The inclusion criteria were as follows: (1) Chinese female adults with lip protrusion who had achieved better sagittal profiles with treatments including extraction of the four first premolars (PM1) without mini-implants. The esthetic outcome analysis was according to complete treatment records (lateral cephalogram, study casts, full-mouth intraoral or panoramic x-rays, and a facial photographic triplet) and judged by the consensus of 2 orthodontists to avoid individual subjective bias; (2) age from 18 to 30 years; (3) ANB > 0°; (4) overall good health; (5) body mass index (BMI) in the range of 18 to 24 kg/m^2^; and (6) no obvious facial asymmetry.

Twenty female subjects (25.5 ± 2.1 years) from among undergraduate students from Peking University, School and Hospital of Stomatology, Beijing, China, were enrolled as the non-treatment group.

The inclusion criteria were as follows: (1) Chinese female adults aged from 18 to 30 years; (2) mild crowding or spacing (< 4 mm); (3) ANB > 0°; (4) overall good health; (5) BMI in the range of 18 to 24 kg/m^2^; and (6) no obvious facial asymmetry.

The exclusion criteria for all subjects were as follows: (1) previous orthodontic treatment; (2) anterior or posterior crossbite; (3) cleft lip and palate or other craniofacial syndromes; and (4) defects of dentition.

**Fig. 1:**
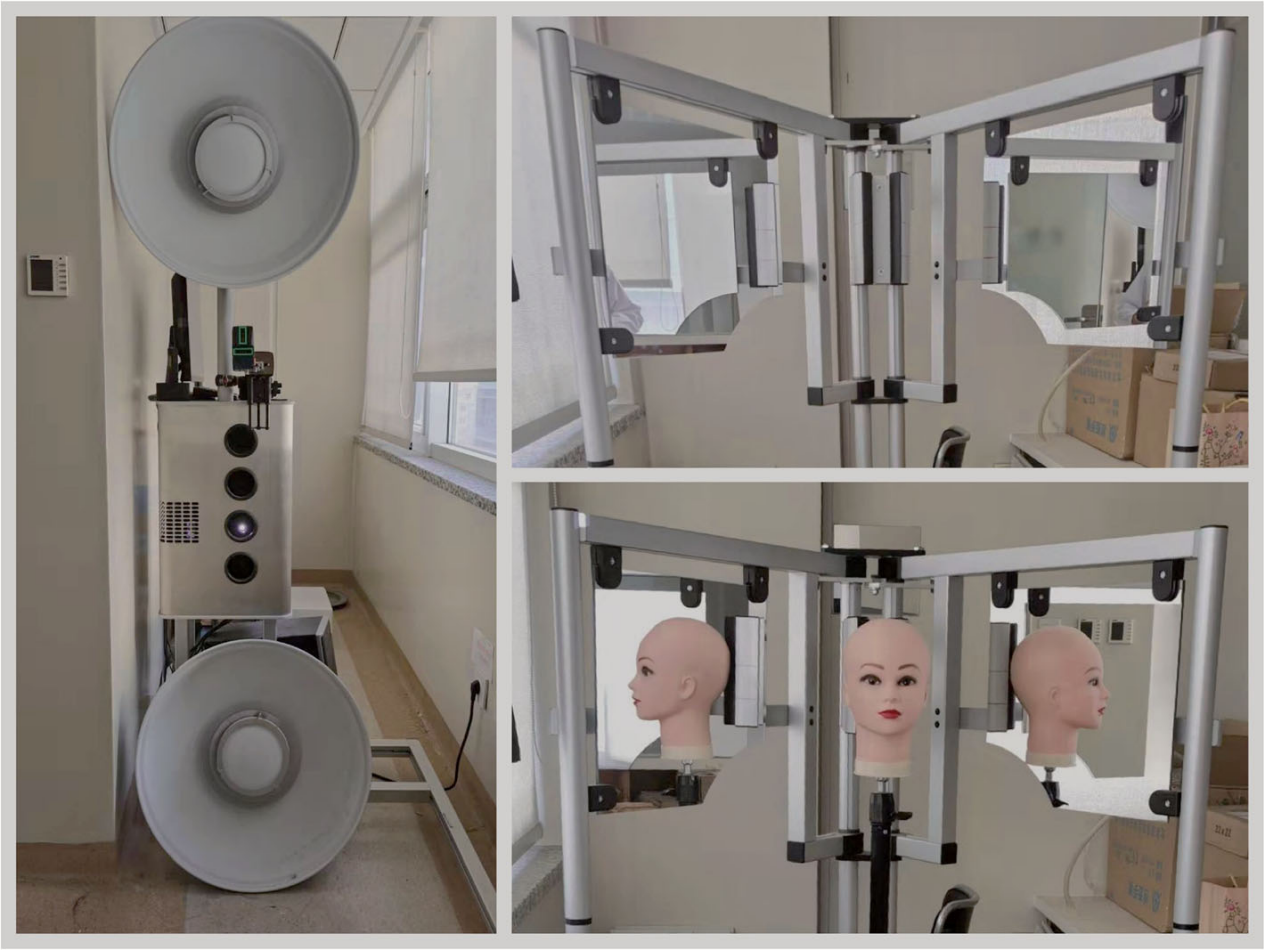
Three-dimensional (3D) facial scanning device

Three-dimensional facial scans (Fig. 1) were available for each subject and were acquired using a structured light-scanning system (accuracy: ±0.05 mm; 1024 pixels × 768 pixels; 3D CaMega; Boweihengxin Technology Inc., Beijing, China). To achieve the rest position of the lips, before starting the scan subjects were asked to relax their lips with full relaxation for 1 or 2 s in a natural head position and then started to pronounce the word “Emma”. Subjects were asked to maintain the pronunciation of ‘mmm’ (during the pronunciation of “Emma”) until the completion of scan. [14]. All subjects had T1 and T2 facial scans. In the treatment group, T1 was the pre-treatment time point, and T2 was the post-treatment time point. In the non-treatment group, the duration between T2 and T1 was at least 12 months.

### Reconstruction and analysis of morphological changes in the lip vermilion

#### Coordinate system construction

**Fig. 2:**
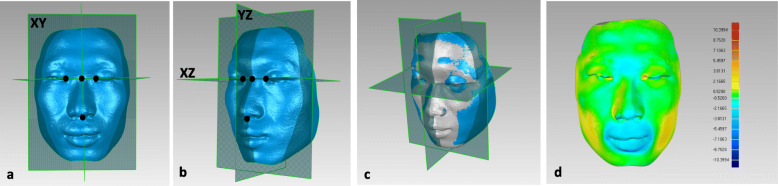
Construction of the coordinate system (**a**, **b**). Superimposition of the two models before (grey) and after (blue) orthodontic treatment with a unified coordinate system (**c**). Color-coded displacement map (blue: inward displacement; green: no change; red: outward displacement) showing the three-dimensional superimposition for the two models (**d**).

The coordinate system (Fig. 2 a and b) was constructed using the method described by Alqattan et al. [15] using the software Geomagic Qualify 12 (3D Systems, Rock Hill, South Carolina). The point located halfway between the inner canthi of the eyes was taken as the origin of the coordinate system at which three planes coincided. The transverse plane (XZ) was constructed to contain the right and left pupils and the soft tissue nasion (N). The sagittal plane (YZ) was set to be the perpendicular plane that contained the N-subnasal (Sn) line as the plane of symmetry of the original mirror face structure. The coronal plane (XY) was perpendicular to the sagittal and transverse planes.

#### Superimposition and visual analysis of morphological changes in the lip vermilion

The two selected digital facial scans (T1 and T2) in the coordinate system were superimposed according to the “best-fit alignment” algorithm [16]; thus, a superimposed 3D image with a unified system was obtained for each subject (Fig. 2 c). Color-coded displacement maps were constructed for visualization of the soft-tissue displacement in the direction of the surface normal (blue: inward displacement; green: no change; red: outward displacement) (Fig. 2d).

#### Quantitative analysis for landmark identification and linear measurements

**Fig. 3:**
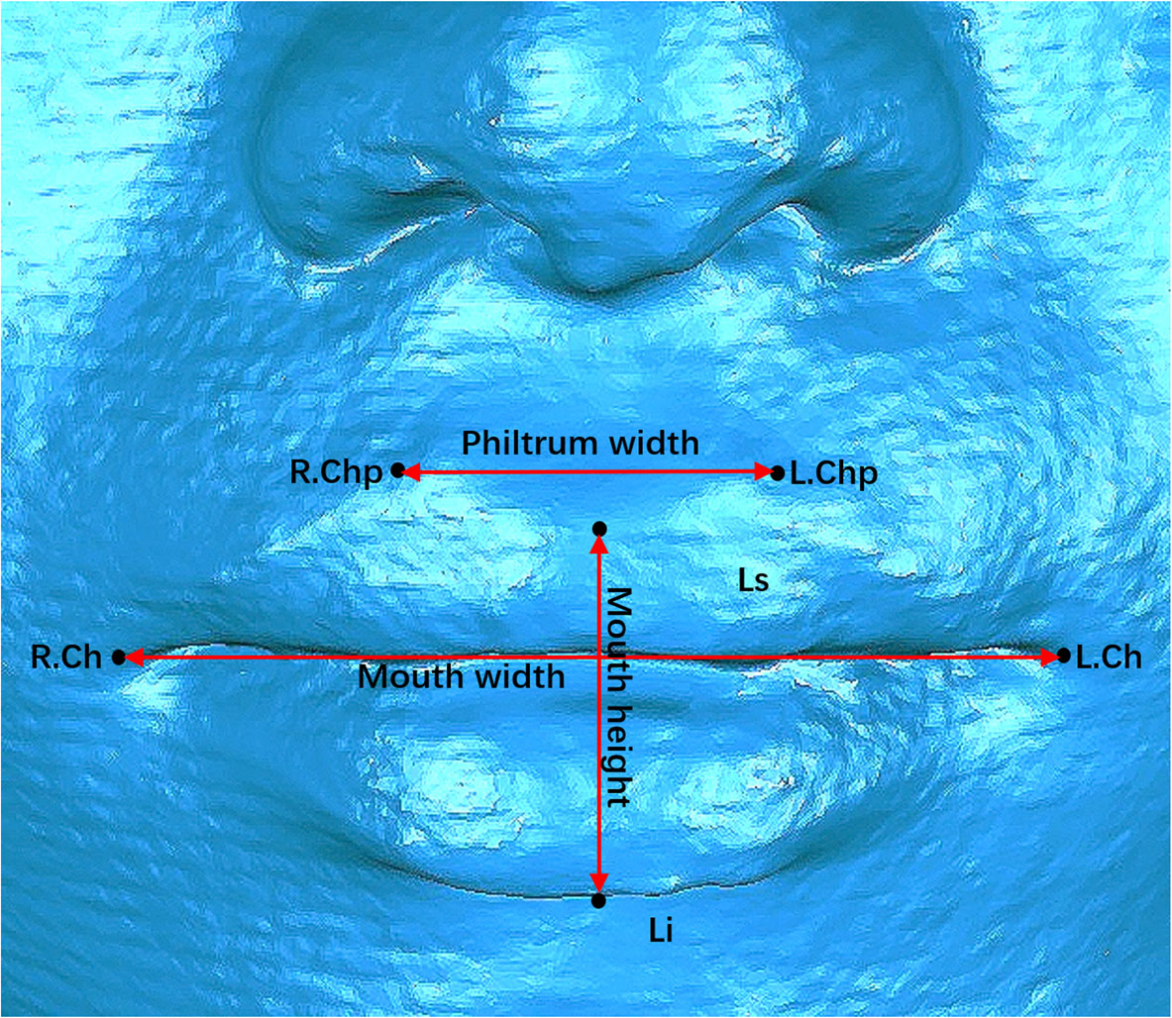
Six facial landmarks (Ls, Ll, R.Chp, L.Chp, R.Ch, and L.Ch) and three linear measurements (mouth height, philtrum width, and mouth width).

Table 1 provides abbreviations and definitions of each lip vermilion measurement [17]. Six facial landmarks (Fig. 3; Table 1) were marked on the 3D image, including two landmarks in the middle line (Ls, Li) and two bilateral landmarks (R.Chp, L.Chp, R.Ch, L.Ch). Three straight line distances (Fig. 3; Table 1) as the linear measurements (mouth height, philtrum width, and mouth width) were measured from 3D images.

**Table 1:** Definition of lip vermilion measurements

Variable	Definition
Landmarks in the midline	
Ls	Labiale superius
Li	Labrale inferius
Bilateral landmarks	
R.Chp (right chresta philtri)	Most prominent point of the vermilion border of right chresta philtri of the upper lip
L.Chp (left chresta philtri)	Most prominent point of the vermilion border of left chresta philtri of the upper lip
R.Ch (right cheilion)	Most lateral extent of the outline of the lip on the right side
L.Ch (left cheilion)	Most lateral extent of the outline of the lip on the left side
Linear measurements (mm)	
Mouth height	Vertical distance between labrale superius (Ls) and labrale inferius (Li)
Philtrum width	Horizontal distance between right and left chresta philtri (Chp)
Mouth width	Horizontal distance between right and left cheilion (Ch)
Area measurements (mm^2^)	
Upper vermilion area	Area of the upper vermilion along the surface
Lower vermilion area	Area of the lower vermilion along the surface
Total vermilion area	Area of the upper and lower vermilion along the surface

#### Set‐up of the measuring planes and quantitative analysis for area and volumetric measurements

**Fig. 4:**
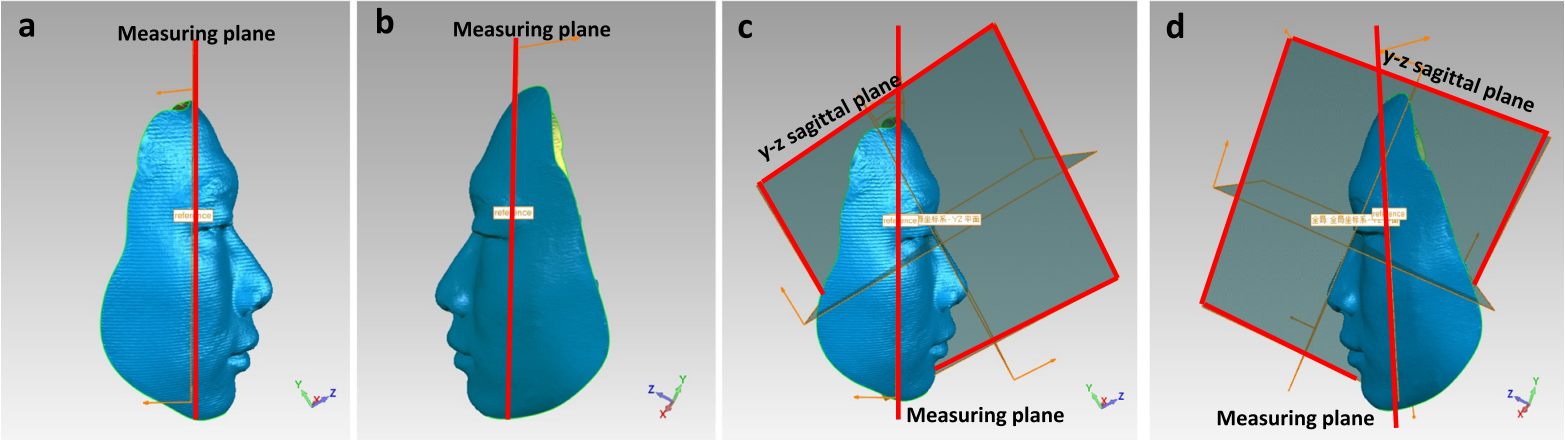
Illustration of the measuring plane (**a**, **b**). Illustration of the measuring plane perpendicular to the YZ sagittal plane and passing through the bilateral inner canthi point (**c**, **d**).

**Fig. 5:**
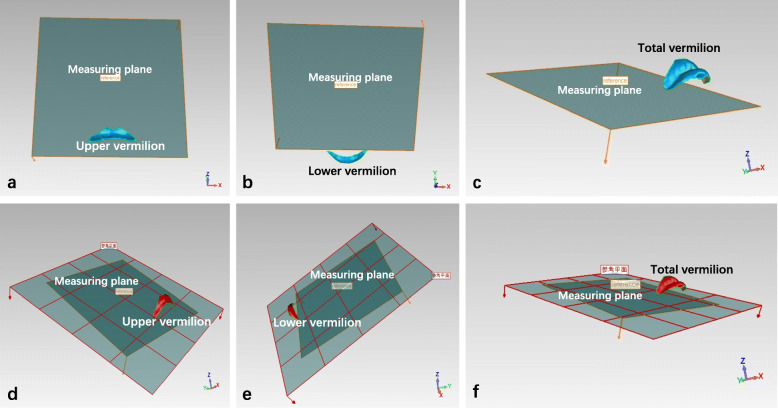
Illustration of the upper, lower, and total vermilion (blue) marginated manually (**a**–**c**). Illustration of the upper, lower, and total vermilion volumes projected to the measuring planes (red) for quantitative analysis (**d**–**f**).

The measuring plane was defined (Fig. 4 a and b) for quantitative analysis. In the unified coordinate system of the superimposed 3D image, the measurement plane (Fig.4 c and d) was perpendicular to the YZ sagittal plane, which passed through the bilateral inner canthi point. The 3D surfaces were marginated manually according to vermilion anatomical morphology (Fig. 5 a–c), the other non-vermilion part was erased [18], and the remainder was projected onto the measurement planes, enabling three area measurements (upper, lower, and total vermilion area along the 3D surface) and three volumetric measurements (upper, lower, and total vermilion volumes projected onto the measurement planes) to be measured (Fig. 5 d-f). The space deviations for volumetric changes (T2 measurement − T1 measurement = space deviation) in the upper, lower, and total vermilion were constructed and used for quantitative analysis.

### Statistical analysis

Data were analysed using SPSS software (version 23.0; IBM Corp., Armonk, NY, USA) and G power (version 3.1.9.4; Germany). All measurements were repeated by the same operator. The degree of intra-observer error was assessed by comparing each measurement with measurements on 20 subjects chosen randomly in an interval longer than two weeks. The threshold for acceptable intra-observer error for each measurement was 0.90 with the calculation of the intraclass correlation coefficient (ICC). The normality of the data from 3D facial scans was confirmed using the Shapiro–Wilk test. A t-test was performed to evaluate changes during orthodontic treatment. A *p*-value of < 0.05 was considered statistically significant.

## Results

In the treatment group, the quantitative analysis results of the changes in landmark identification pre-treatment and post-treatment are shown in Table 2. There were no significant changes (*p* > 0.05) in any of the assessments in the vertical dimension among all landmarks. There were significant changes in the horizontal dimension of the points R.Chp (*p* < 0.01) and L.Chp (*p* < 0.05). There were significant changes in the sagittal dimension of the points Li (*p* < 0.01), R.Ch (*p* < 0.05), and L.Ch (*p* < 0.01).

**Table 2:** Comparison of three-dimensional changes in soft tissue landmarks in the treatment group

	Horizontal dimension		Vertical dimension		Sagittal dimension	
Landmark	change	*p*-value	change	*p*-value	change	*p*-value
Ls	0.48 ± 2.79	0.437(NS)	-1.24 ± 10.69	0.598(NS)	-2.30 ± 9.53	0.272(NS)
Ll	0.22 ± 2.70	0.712(NS)	1.63 ± 14.39	0.565(NS)	-7.21 ± 11.16	0.004**
R.Chp	2.10 ± 3.26	0.004**	-1.55 ± 10.18	0.487(NS)	-1.54 ± 8.68	0.417(NS)
L.Chp	-1.79 ± 3.18	0.011*	-1.51 ± 10.16	0.499(NS)	-1.51 ± 8.65	0.428(NS)
R.Ch	0.84 ± 3.61	0.292(NS)	-0.98 ± 11.45	0.695(NS)	-3.65 ± 6.82	0.016*
L.Ch	0.50 ± 3.25	0.488(NS)	-0.66 ± 11.41	0.791(NS)	-4.69 ± 7.16	0.004**

Values are presented as the mean ± standard deviation (mm). The change is the difference between post-treatment and pre-treatment values. A paired t-test was used to compare pre- and post-orthodontic treatment in each group. NS, non-significant; *p<; 0.05; **p <; 0.01.

The quantitative analysis results of the changes in the linear and area measurements pre-treatment versus post-treatment in the treatment group are shown in Table 3. Table 4 shows a comparison of the changes in the linear and area measurements between T1 and T2 in the non-treatment group. And there were no significant differences both in the linear and area measurements in the non-treatment group. Table 5 shows a comparison between the treatment group and the non-treatment group. For linear measurements in the treatment group, philtrum width and mouth height decreased significantly during orthodontic treatment (*p* < 0.01 and *p* < 0.05, respectively), and the mean differences in each from T1 to T2 were significantly different compared with the non-treatment group (*p* = 0.01 and *p* < 0.05, respectively). No significant change was observed in mouth width in the treatment group. There were no significant differences in the area measurements of the upper vermilion in the treatment group, while the lower vermilion area and total vermilion area were significantly decreased after orthodontic treatment (*p* < 0.01 and *p* < 0.05, respectively) and the mean differences in each were significantly different between the treatment and the non-treatment groups (*p* < 0.05 and *p* < 0.05, respectively).

**Table 3:** Comparison of the changes in the linear and area measurements pre- and post-treatment in the treatment group

Variable	Pre-treatment	Post-treatment	*p*-value
Linear measurements (mm)			
Philtrum width	16.69 ± 2.05	15.76 ± 1.89	0.008^**^
Mouth width	50.51 ± 4.14	50.83 ± 3.99	0.488(NS)
Mouth height	21.89 ± 2.94	20.81 ± 2.36	0.027^*^
Area measurements (mm^2^)			
Upper vermilion area	683.63 ± 156.50	677.38 ± 128.56	0.752(NS)
Lower vermilion area	638.43 ± 125.58	587.43 ± 138.48	0.003^**^
Total vermilion area	1200.09 ± 196.62	1130.27 ± 243.08	0.031^*^

Values are presented as the mean ± standard deviation. A paired t-test was used to compare pre- and post-orthodontic extraction in each group. *NS* non-significant; *p < 0.05; **p < 0.01

**Table 4:** Comparison of the changes in the linear and area measurements between T1 and T2 in the non-treatment group

Variable	T1	T2	*p*-value
Linear measurements (mm)			
Philtrum width	15.97 ± 1.64	15.96 ± 1.47	0.792(NS)
Mouth width	47.50 ± 2.50	47.51 ± 2.38	0.740(NS)
Mouth height	20.67 ± 1.16	20.77 ± 1.21	0.688(NS)
Area measurements (mm^2^)			
Upper vermilion area	687.78 ± 84.19	691.77 ± 105.82	0.591(NS)
Lower vermilion area	620.16 ± 96.65	628.83 ± 107.00	0.623(NS)
Total vermilion area	1132.73 ± 110.01	1214.60 ± 185.97	0.221(NS)

Values are presented as the mean ± standard deviation. An independent t-test was used to compare the treatment and non-treatment groups. *NS* non-significant; *p < 0.05; **p < 0.01

**Table 5:** Mean differences from T1 to T2 in the linear and area measurements between the treatment and non-treatment groups

Variable	Treatment group	Non-treatment group	*p*-value
Linear measurements (mm)			
Philtrum width	− 0.94 ± 2.17	− 0.01 ± 0.37	0.010^*^
Mouth width	0.32 ± 3.00	0.01 ± 0.59	0.516(NS)
Mouth height	− 1.08 ± 3.06	0.10 ± 0.32	0.017^*^
Area measurements (mm^2^)			
Upper vermilion area	− 6.25 ± 127.37	34.00 ± 81.61	0.139(NS)
Lower vermilion area	− 51.01 ± 104.88	8.67 ± 87.21	0.023^*^
Total vermilion area	− 69.81 ± 202.98	52.12 ± 156.48	0.012^*^

Values are presented as the mean ± standard deviation (mm^3^). An independent t-test was used to compare the treatment and non-treatment groups. *NS* non-significant; *p < 0.05; **p < 0.01

**Fig. 6:**
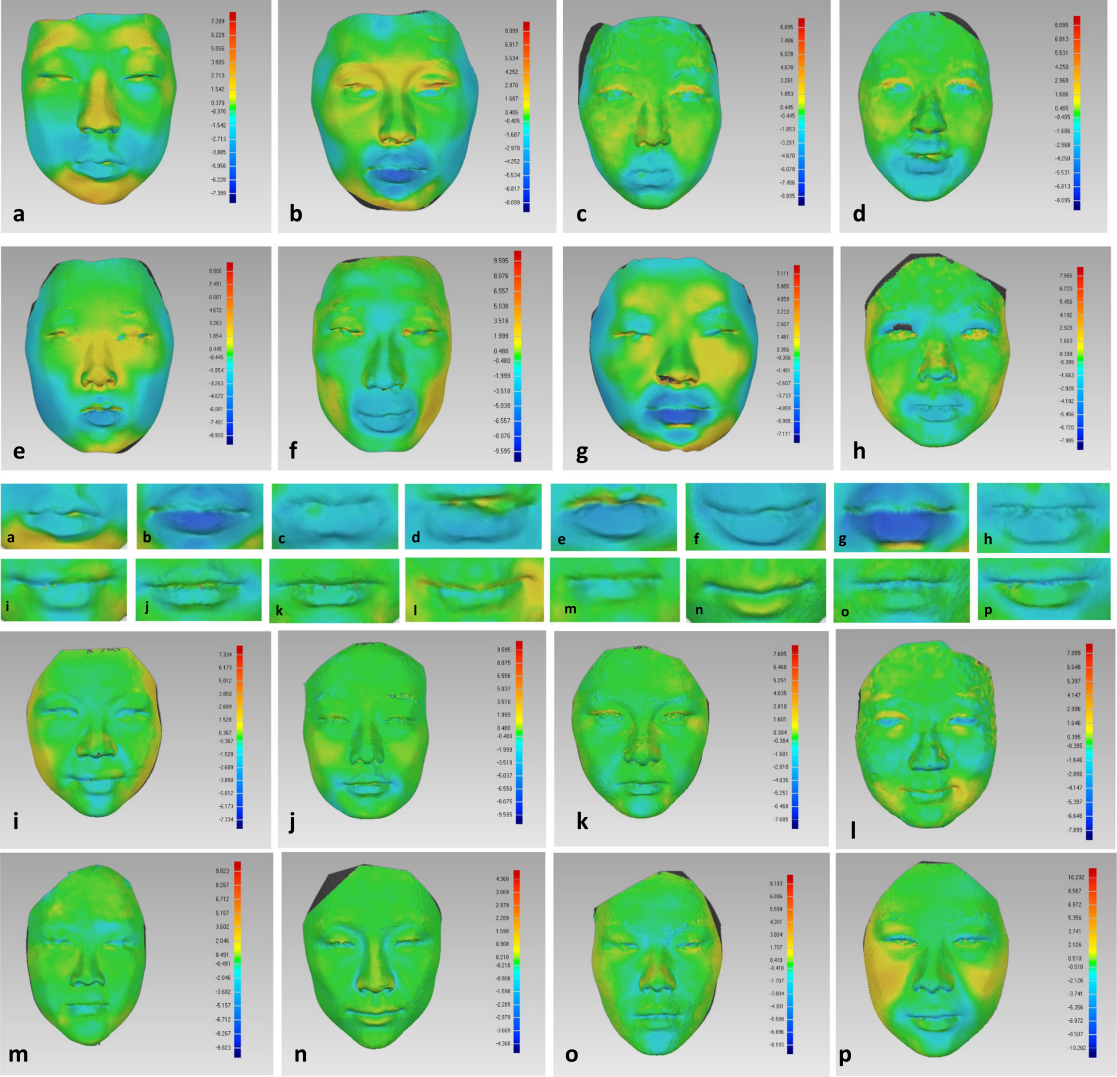
Color-coded displacement map showing individual variability in facial soft tissues among eight subjects in the treatment group (**a**–**h**) and lip vermilions (**a**–**h**). Color-coded displacement map showing individual variability in facial soft tissue among eight subjects in the non-treatment group (**i**–**p**) and lip vermilions (**i**–**p**). Blue: inward displacement; green: no change; red: outward displacement.

As shown in Fig. 6, color-coded displacement maps of visual analysis were constructed to assess morphological changes in the lip vermilion during orthodontic therapy in the treatment group (Fig. 6 a–h) and morphological changes in the non-treatment group between T2 and T1 (Fig. 6 i and p). Table 6 shows the comparison of volumetric changes between the treatment and non-treatment groups in the upper, lower, and total vermilion; there were significant differences in the three volumetric measurements (*p* < 0.05, *p* < 0.01, and *p* < 0.01, respectively).

**Table 6:** Volumetric changes between the treatment and non-treatment groups

Variable	Treatment group	Non-treatment group	*p*-value
Upper vermilion volume	− 1625.81 ± 2607.15	280.51 ± 2667.69	0.012^*^
Lower vermilion volume	− 1893.22 ± 2196.20	495.16 ± 2568.11	0.001^**^
Total vermilion volume	− 2933.14 ± 5478.74	1158.99 ± 4654.83	0.004^**^

Values are presented as the mean ± standard deviation (mm3). An independent t-test was used to compare the treatment and non-treatment groups. *NS* non-significant; *p < 0.05; **p < 0.01.

A post hoc analysis for the t-test (sample size group 1 = 42; sample size group 2 = 20; significance level α = 0.05; effect size = 0.80) was computed and showed that the statistical power was 0.83. The intra-observer reliability was estimated using the ICC for each measurement in three dimensions. In summary, the ICC values indicated excellent reliability (> 0.90 for all calculations).

## Discussion

At present, most research methods used to measure the soft tissue morphology of the lips are based on 2D films, and the measurement indicators are not sufficiently comprehensive. This is because the facial soft tissue is an irregular curved surface. In many cases, soft tissue samples must reflect 3D features, such as curved surfaces, to enable a more accurate assessment of morphology. In this study, 3D facial scanning technology was used to establish an appropriate measurement method. The data collected using a 3D facial scanner were analysed using Geomagic Qualify software and were easy to use to quantify and describe morphological changes in the lip vermilion by establishing a coordinate system.

Many factors such as masticatory muscle tension and physiological conditions of the lip (e.g., thin/thick type and muscle tension) might influence the reproducibility of lip position and contribute to variations in measurements of changes in the vermilion [14]. In this study, adult female subjects who did not receive orthodontic treatment were enrolled as the non-treatment group. By comparing this group with the non-treatment group, statistically significant differences in the measurements in the treatment group were mainly due to the influence of extraction treatment and less likely to be affected by other influencing factors.

In the present study, the statistical results of the linear and area measurements were considered in combination with the results of landmark identification for a preliminary exploration of the pattern of morphological changes in the lip vermilion after extraction treatment. There was no significant change in the mouth width, which may be related to the observation that R.Ch and L.Ch showed no significant changes in their horizontal dimensions. The upper vermilion area was not significantly different, which may be associated with the fact that R.Chp, L.Chp, and Ls did not show significant changes in their sagittal dimensions. The mouth width and upper vermilion area appeared to be stable after the extraction treatment. The significant decrease in the philtrum width may be related to the significant changes in R.Chp and L.Chp in the horizontal dimension. The lower vermilion area was significantly reduced, which may be associated with significant changes in R.Ch, L.Ch, and Li in the sagittal dimension. The significant decrease in the mouth height may be related to significant changes in Li in the vertical dimension. Trisnawaty et al. reported that the mouth height decreased by 0.39 mm according to the linear measurement of St–Me when the four first premolars were extracted [19]. However, Maltagliati et al. reported that the lower lip height increased (4.61 ± 3.61 mm) significantly, and the vertical dimension of the upper lip was not modified in the group treated with extraction [20], which indicates that mouth height was increased in their study. The controversy presented by these articles requires further investigation.

A qualitative impression was obtained using the color-coded displacement map. As presented in Fig. 6, the vermilion area in the treatment group retracted backwards (shown as blue colour presenting inward displacement) after orthodontic treatment and differed from the superimposed results observed in the non-treatment group. A statistically significant degree of retraction occurred in the vermilion (p < 0.01) after orthodontic extraction in the present study, which can be used as quantitative evidence to support the qualitative results of the color-coded displacement map. Both the upper (p < 0.05) and lower (p < 0.01) vermilion were retracted in the treatment group. These results are in accordance with the clinical observations of historical studies [4, 21]. Lee et al. reported that extraction of the four premolars led to significantly greater retraction of the upper/lower lip from Ricketts’ E plane [22] Liu et al. reported a significantly smaller lip vermilion after extraction, indicating that the lip vermilion was more slender when the mouth height–width ratio was decreased [10]. Nevertheless, contradicting conclusions drawn by Freitas et al. [23], and Ayhan Basciftci et al. [24] reported that extraction of the first premolars did not imply a greater degree of retraction in the lip soft tissue. A more harmonious vermilion was considered attractive and had positive effects on lip aesthetics [5], but the relationship between lip morphology and facial aesthetics is complex. Ethnicity, skin colour, age, and profile characteristics might all affect the quality of treatment [25]. According to Kocadereli et al., for the sake of retraction of the upper and lower lips, extraction caused a harmful effect on facial aesthetics, flattening the facial profile [26]. However, in cases of nose or chin protrusion, compensatory lip protrusion improves the profile attractiveness [27]. The area and volume of the lower vermilion after extraction had a greater degree of retraction than those of the upper vermilion retraction, suggesting that the lower vermilion plays a major role in retraction. These results are consistent with historical articles that studied the responding patterns of soft tissue. A recent systematic review reported that the average lip retraction after extraction of the 4 premolars was 1.4 mm for the upper vermilion and 2.0 mm for the lower vermilion [28]. The difference between the upper and lower vermilions may vary considerably with incisor variables [29], the distance of the mandibular lip to the aesthetic line [5], and other factors.

One limitation of the present study was the small sample size, and there was remarkable variation in lip morphology among different populations between races and ethnic groups. López-Mateos, M. M et al. reported that in southern Spain European female adults with normal occlusion presented narrower philtrum width (9.29 ± 1.95 mm) and shorter vermilion height (11.83 ± 2.45 mm) and similar mouth width (47.34 ± 3.65 mm) [25]. According to Othman et al., female Malaysians also presented shorter philtrum width (10.40 ± 1.14 mm) and similar mouth width (48.00 ± 2.61 mm) [30]. But Kim S Y et al. reported that young adult women in Korea showed narrower inter-cupid bow width (3.29 ± 0.42 mm) and narrower facial mouth width (32.36 ± 1.97 mm) [8]. Previous 3D studies found that the mouth height of Asian adult females was in the range of 16.0 mm to 19.0 mm [10, 31, 32], and the results in the present study were larger both pre- and post-treatment in the treatment group. Although samples were enrolled in an effort to eliminate bias, heterogeneous intervention could not be avoided, which is a common problem in clinical research. Large standard deviations among people can provide evidence, and the standard deviations of the upper, lower and total vermilion, as area measurements, were larger than 80 mm^2^ in all the calculations in this study.

Thus, this study was a preliminary exploration, and the results should not be generalized to clinical orthodontic treatment as a whole (e.g., in the context of different malocclusions or different treatment methods). A larger sample size would be needed in further studies to clarify the findings. Possible influencing factors that could contribute to changes in the vermilion (e.g., facial soft tissue conditions and skeletal patterns) also require further investigation or verification.

## Conclusions

The present study established a method to quantify and qualify possible changes in the lip vermilion during orthodontic extraction in female adult patients. The philtrum width, mouth height and lower vermilion area appeared to decrease, and the mouth width and upper vermilion area appeared to be stable after the extraction treatment. Significant retraction of the lip vermilion was observed, and the lower vermilion area showed a greater degree of retraction than the upper vermilion. Further studies with a larger sample size and a multitude of parameters would help to verify the results of the present study.

## Data Availability

The datasets used and/or analyzed during the current study are available from the corresponding author on reasonable request.

## Abbreviations

BMI: Body mass index
3D: Three-dimensional
PM1: First premolar
Ls: Labiale superius
Li: Labrale inferius
R.Chp: Right chresta philtri
L.Chp: Left chresta philtri
R.Ch: Right cheilion
L.Ch: Left cheilion
ICC: Intraclass correlation coefficient
NS: Non-significant
